# *eNOS* polymorphisms as predictors of efficacy of bevacizumab-based chemotherapy in metastatic colorectal cancer: data from a randomized clinical trial

**DOI:** 10.1186/s12967-015-0619-5

**Published:** 2015-08-11

**Authors:** Paola Ulivi, Emanuela Scarpi, Alessandro Passardi, Giorgia Marisi, Daniele Calistri, Wainer Zoli, Marzia Del Re, Giovanni Luca Frassineti, Davide Tassinari, Stefano Tamberi, Bernadette Vertogen, Dino Amadori

**Affiliations:** Biosciences Laboratory, Istituto Scientifico Romagnolo per lo Studio e la Cura dei Tumori (IRST) IRCCS, Via Maroncelli 40, 47014 Meldola, Italy; Unit of Biostatistics and Clinical Trials, IRST IRCCS, Meldola, Italy; Department of Medical Oncology, IRST IRCCS, Meldola, Italy; Department of Clinical and Experimental Medicine, Pisa University Hospital, Pisa, Italy; Department of Oncology, Per gli Infermi Hospital, Rimini, Italy; Oncology Unit, Degli Infermi Hospital, Faenza, Italy

**Keywords:** Advanced colorectal cancer, Bevacizumab, eNOS, VEGF, SNPs

## Abstract

**Background:**

Bevacizumab plus chemotherapy is a widely used therapeutic option for first-line treatment of metastatic colorectal cancer (mCRC). However, molecular predictors of bevacizumab efficacy have not yet been identified. We analyzed vascular endothelial growth factor (*VEGF*) and endothelial nitric oxide synthase (*eNOS*) polymorphisms in relation to response to bevacizumab.

**Methods:**

Two hundred and thirty-seven patients with mCRC enrolled onto the phase III prospective multicentre randomized “Italian Trial in Advanced Colorectal Cancer (ITACa)” trial were evaluated. One hundred fourteen patients received chemotherapy plus bevacizumab (CT + B) and 123 received chemotherapy (CT) alone. Five single nucleotide polymorphisms (SNPs) (−2578, −1498, −1154, −634 and +936) for *VEGF* and 2 SNPs (−786, +894) and one variable number tandem repeat in intron 4 for *eNOS* were analyzed for each patient. The polymorphisms were assessed in relation to progression-free survival (PFS), objective response rate (ORR) and overall survival (OS).

**Results:**

*VEGF* 936C/T, *eNOS* +894 G/T and VNTR were significantly correlated with outcome in CT + B patients, but not in CT-only patients. In particular, patients with a specific haplotype combination of the 2 *eNOS* polymorphisms (defined *eNOS* Haplo1/Haplo1 and *eNOS* Haplo 2/Haplo2) showed significantly longer PFS (15.0 vs 9.1 months, *P* = 0.001) and OS (34.5 vs 20.5 months *P* = 0.002), and a higher ORR (71 vs 45.9%, *P* = 0.013) than those with the other genotypes, respectively.

**Conclusions:**

Specific *eNOS* polymorphisms may be capable of identifying a subset of mCRC patients who are more responsive to bevacizumab-based chemotherapy. If confirmed, these results would permit individually tailored treatment with bevacizumab.

**Electronic supplementary material:**

The online version of this article (doi:10.1186/s12967-015-0619-5) contains supplementary material, which is available to authorized users.

## Background

The therapeutic approach to metastatic colorectal cancer (mCRC) has changed in recent years, mainly thanks to the introduction of biologic drugs such as cetuximab, a monoclonal antibody (MoAb) directed against the epidermal growth factor receptor (*EGFR*), or bevacizumab, a MoAb that blocks the vascular endothelial growth factor (*VEGF*) [1]. As *RAS*-mutated patients show no benefit from anti-EGFR therapy, *RAS* mutations are used as a marker to select candidates for cetuximab treatment [2, 3]. However, they are not predictive of the efficacy of bevacizumab [4, 5], for which there are still no known biomarkers that are capable of distinguishing between responsive and non responsive patients. The correct selection of patients to be treated with bevacizumab-based chemotherapy could allow for the drug only to be given to those patients who will really benefit from it and for a reduction in the number of adverse effects.

Some studies have reported that specific *VEGF* single nucleotide polymorphisms (SNPs) would seem to affect gene transcription, with a consequent variation in VEGF expression [6, 7]. Other studies have evaluated the role of *VEGF* SNPs in relation to response to bevacizumab [8–12], the contradictory results reported possibly due to different study designs. In a retrospective study by Loupakis and colleagues, *VEGF* −1498 C/T variants proved capable of predicting response to bevacizumab plus FOLFIRI [11]. Similarly, in a more recent study [10], the same variants together with *VEGF* −2578 C/A were shown to predict response to bevacizumab treatment. A prospective study by Koutras and colleagues reported that only the *VEGF* −1154 G/A variants were associated with response and overall survival (OS) in patients treated with bevacizumab plus FOLFIRI or XELIRI [9]. The same *VEGF* SNPs were associated with progression-free survival (PFS) in another study [8] in which *VEGF* −634 G/C was associated with response. These SNPs have also been associated with cardiovascular adverse effects induced by bevacizumab, in particular, hypertension [10], and a correlation has been reported between response and bevacizumab-induced hypertension [13]. Recently, another study found that *VEGF* and endothelial nitric oxide synthase (*eNOS*) polymorphisms were associated with sunitinib-induced hypertension in patients with metastatic renal cancer, with grade three hypertension identified as an independent predictor of OS [14].

In the present study, we analyzed *VEGF* and *eNOS* polymorphisms in relation to clinical outcome (PFS, overall response rate [ORR] and OS) in mCRC patients undergoing bevacizumab-based chemotherapy in the phase III prospective multicentre randomized “Italian Trial in Advanced Colorectal Cancer (ITACa)” (EudraCT no. 2007-004539-44 and on ClinicalTrials.gov (NCT01878422).

## Methods

### Case series

The study was approved by the Local Ethics Committee (Comitato Etico Area Vasta Romagna e IRST) and informed consent was obtained from all patients before blood samples were obtained for genotype testing. Participation in the ITACa biological study was not mandatory for those taking part in the clinical trial. Of the 376 patients with mCRC enrolled onto the ITACa trial, 237 had sufficient biological material archived to be considered for this planned secondary analysis.

Inclusion criteria, patient characteristics, randomization strategy and the clinical results of the ITACa study are reported elsewhere [15]. Patients were randomized to receive first-line chemotherapy (CT) (FOLFOX4 or FOLFIRI) only or CT plus bevacizumab (B). FOLFOX4 consisted of oxaliplatin 85 mg/m^2^ as a 2-h infusion on day one and leucovorin 100 mg/m^2^ as a 2-h infusion followed by bolus 5-FU 400 mg/m^2^ and a 22-h infusion of 5-FU 600 mg/m^2^ on days 1–2 every 2 weeks. FOLFIRI consisted of the same 5-FU +leucovorin regimen with the addition of irinotecan 180 mg/m^2^ as a 90-min infusion on day one. B was administered as a 30- to 90-min intravenous infusion at a dose of 5 mg/kg on day 1 of each 2-week cycle. Treatment was to be continued until progressive disease (PD), withdrawal of consent or unacceptable toxicity, whichever came first. Tumor assessment tests were performed within 28 days of starting the study treatment and repeated every 8 weeks during treatment until PD.

The clinical characteristics of patients are represented in Table 1. One hundred and fourteen patients received CT + B and 123 patients received CT only (control group). We considered *KRAS* status as an independent variable because it was a stratification factor in the clinical study. Furthermore, to our knowledge, there are no significant data in the literature that attest to the fact that KRAS plays a role in response to bevacizumab.

**Table 1:** Patient Characteristics

Variable	CT + B (n = 114) n (%)	CT (n = 123) n (%)
Median age, years (range)	66 (34–83)	67 (37–82)
Gender		
Male	70 (61.4)	74 (60.2)
Female	44 (38.6)	49 (39.8)
Performance status (ECOG)		
0	97 (85.1)	102 (82.9)
1	17 (14.9)	21 (17.1)
Tumor localization		
Rectum	31 (27.2)	34 (27.6)
Colon	83 (72.8)	89 (72.4)
Histology		
Adenocarcinoma	104 (91.2)	119 (96.8)
Mucinous adenocarcinoma	10 (8.8)	4 (3.2)
Grade		
1	0	0
2	56 (64.4)	67 (65.1)
3	31 (35.6)	36 (34.9)
Unknown	27	20
Stage at diagnosis		
I–III	29 (26.4)	31 (27.2)
IV	81 (73.4)	83 (72.8)
Unknown	4	9
Chemotherapy regimen planned		
FOLFOX4	69 (60.5)	73 (59.4)
FOLFIRI	45 (39.5)	50 (40.6)
*KRAS* status^a^		
Wild type	67 (59.8)	69 (58.5)
Mutated	45 (40.2)	49 (41.5)
Unknown	2	5
Prior cancer therapy		
Surgery	88 (77.2)	91 (74.0)
Radiotherapy	11 (9.6)	11 (8.9)
Adjuvant chemotherapy	18 (15.8)	17 (13.8)

^a^Mandatory as consequence of amendment n.1 of 3 May 2009.

All patients were evaluated for response [according to Response Evaluation Criteria in Solid Tumors (RECIST) guidelines], PFS and OS. In particular, response was classified as complete response (CR), partial response (PR), stable disease (SD) or PD, and patients with CR or PR were defined as responsive.

### Genomic DNA extraction

Peripheral blood samples were available for polymorphism analysis in 153 patients, whereas only paraffin-embedded tumor samples were available for 84 patients.

Genomic DNA was extracted from whole blood using QIAamp DNA Minikit (Qiagen SPA, Milan, Italy) following the manufacturer’s protocol. DNA was extracted from formalin-fixed paraffin- embedded (FFPE) tumor tissue starting from 5-μM FFPE tissue sections. Tissue was lysed in 50 mM of KCl, 10 mM of Tris–HCl pH 8.0, 2.5 mM of MgCl2 and Tween-20 0.45%, supplemented with Proteinase K at a concentration of 1.25 mg/ml, overnight at 56°C. Proteinase K was inactivated at 95°C for 10 min, after which samples were centrifuged twice to eliminate debris. Supernatant was assessed for DNA quality and quantity by Nanodrop (Celbio Spa, Milan, Italy) and then underwent molecular analysis.

### Genotyping analyses

**Fig. 1:**
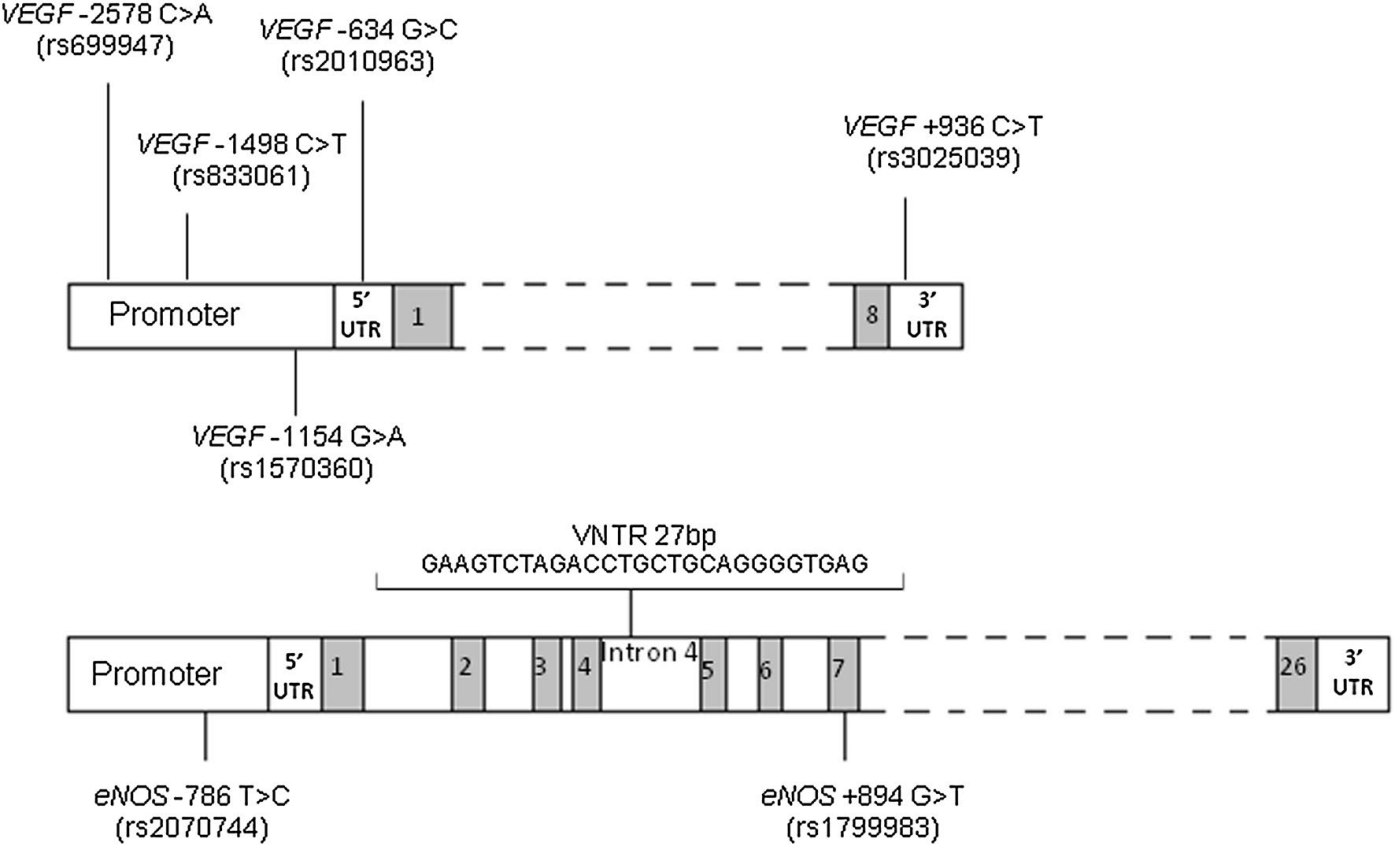
*VEGF* and *eNOS* polymorphisms (with rs reference numbers) analyzed in the study.

Genotyping was performed for five *VEGF* SNPs (*VEGF* −2578C>A, −1498C>T, −1154G>A, −634C>G, +936C>T) and for two SNPs (*eNOS* −786T>C, +894G>T) and one variable number tandem repeat (VNTR) of 27 nucleotides for *eNOS*. The localizations and refSNP (rs) numbers of the different polymorphisms are shown in Fig. 1. All but *eNOS* −786 were analyzed by direct sequencing. The primer sequences and PCR conditions are reported in an Additional file 1. PCRs were performed starting from 50 ng of genomic DNA. *eNOS*-786 was analyzed by Real-Time PCR using a TaqMan SNP Genotyping assay (Assay ID C_15903863_10, Applied Biosystems, Foster City, CA, USA) and starting from 10 ng of DNA.

### Statistical analysis

All polymorphisms were examined for deviation from Hardy–Weinberg equilibrium (HWE) by comparing actual allelic distributions with those expected from HWE using a χ^2^ test. Lewontin’s standardised disequilibrium coefficient (D′) among investigated polymorphisms was assessed using the HaploView programme [16]. The software provides the D′ coefficient as a measure of the non-random association of alleles at different loci. D′ coefficient is equal to one only if two SNPs have not been separated by recombination (or recurrent mutation) during the history of the sample [complete degree of linkage disequilibrium (LD)]. The same software was used to perform haplotype analysis.

The primary aim of the ITACa study was PFS. Secondary efficacy endpoints were ORR and OS. PFS was calculated from the date of randomization to the date of the first observation of disease progression or last follow-up or death in the absence of progressive disease. OS was calculated from the date of randomization to the date of death from any cause, or last follow-up. PFS, OS and their 95% confidence intervals (95% CI) were estimated using the Kaplan–Meier method.

SNPs and haplotypes and clinical outcomes were analyzed separately in each treatment group (CT + B and CT). Adjusted HRs by baseline characteristics (gender, age, performance status, *KRAS* status, tumor localization (rectum/colon) and chemotherapy regimen (FOLFOX4/FOLFIRI) were calculated using the Cox proportional-hazard model. Two-sided 95% CIs for each HR were provided. Covariate selection was based on a list of probable prognostic factors from the ITACa study [15]. Associations between polymorphisms or haplotypes and ORR (CR + PR) were analyzed by logistic regression models. Odds ratios (OR) and 95% CI were adjusted for gender, age, performance status, *KRAS* status, tumor localization (rectum/colon) and chemotherapy regimen (FOLFOX4/FOLFIRI).

All *P* values were based on two-sided testing; to mitigate the issue of multiple testing, a false discovery rate (FDR) of less than 10% was used to determine polymorphisms or haplotypes associated with PFS, OS and ORR. FDR was controlled using the Benjamini-Hochberg step-up procedure [17]. The effect of the interaction between SNPs or haplotypes and treatment on PFS was evaluated using Cox regression models of the entire population (CT + B and CT arms) that included SNPs or haplotypes, treatment and treatment-by-SNPs or -haplotypes. Statistical analyses were performed using SAS Statistical software version 9.3 (SAS Inc., Cary, NC, USA).

## Results

We previously demonstrated that *VEGF* and *eNOS* polymorphism analysis showed concordant results regardless of starting material used (FFPE or blood sample) in all but the *VEGF* −1154G>A SNP [18]. On the basis of this observation, *VEGF* −1154G>A was only analyzed in patients for whom peripheral blood samples were available (153 patients).

The observed allele distributions of *VEGF* −2578, −1498, −1154, −634, +936 polymorphisms and *eNO*S +894, −786, VNTR 4a/b are shown in an Additional file 2; all were in HWE (*P* = 0.95; *P* = 0.95, *P* = 0.12, *P* = 0.30, *P* = 0.91, *P* = 0.50, *P* = 0.85, *P* = 0.26 respectively).

In accordance with the primary endpoint of the ITACA trial, we analyzed the association between the different polymorphisms and PFS in the CT + B and CT only groups. At a median follow-up of 36 months (range 1–65), there had been 103 (90.4%) and 115 (93.5%) progressions and 82 (71.9%) and 91 (74.0) deaths in the CT + B and CT groups, respectively.

In the CT + B group, only the *VEGF* +936 T/C polymorphism was significantly associated with PFS; in particular, the *VEGF* +936 TT genotype was associated with a shorter median PFS compared with other genotypes (7.8 months, 95% CI 1.7–9.1 vs. 10.2 months, 95% CI 9.0–12.4, *P* = 0.036) (Table 2). No statistically significant differences were observed in the CT-only group (Table 2). Of note, the p value did not reach the *P* value threshold adjusted for multiple testing, possibly due to the small number of patients (3) with a *VEGF* +936 TT genotype. *eNOS* +894 G/T was also associated with PFS, and patients with *eNOS* +894 G/T showed a shorter PFS (8.9 months, range 6.8–10.2 months) than those with *eNOS* +894 GG/TT (11.9 months, range 9.6–14.1 months, *P* = 0.013) in the CT + B arm (Table 2; Additional file 3: Figure S1, panel A). This polymorphism was statistically significant after FDR correction. Conversely, no differences were observed in the control group (Table 2, Additional file 3: Figure S1, panel B). Moreover, *eNOS* VNTR 4a/b was associated with PFS in the CT + B arm. Patients homozygous for the five repetitions (VNTR 4bb) showed a longer PFS (10.9 months, range 9.1–12.9 months) than those with other genotypes (9.1 months, range 6.2–11.3 months, *P* = 0.034) (Table 2; Additional file 3: Figure S1, panel C). No statistically significant differences were observed in the control group (Table 2, Additional file 3: Figure S1, panel D).

**Table 2:** Association between *VEGF* and *eNOS* polymorphisms and progression–free survival (PFS)

Polymorphisms	CT + B			CT		
	Median PFS (95% CI)	HR (95% CI)	P*	Median PFS (95% CI)	HR (95% CI)	P*
*VEGF* −2578						
AA	9.3 (3.1–12.5)	1.18 (0.72–1.93)		9.1 (7.8–11.4)	1.27 (0.81–1.99)	
CC/CA	10.4 (8.9–12.4)	1.00	0.515	9.0 (7.8–10.3)	1.00	0.304
*VEGF* −1498						
CC	9.1 (6.4–12.5)	1.30 (0.81–2.11)		9.1 (7.8–11.4)	1.21 (0.77–1.90)	
TT/CT	10.7 (9.1–12.4)	1.00	0.277	9.0 (7.8–10.3)	1.00	0.406
*VEGF* −1154						
AA	11.5 (2.3–18.5)	1.09 (0.54–2.21)		9.1 (3.1–12.2)	1.74 (0.89–3.38)	
GA/GG	10.3 (8.3–12.9)	1.00	0.808	9.6 (8.9–11.3)	1.00	0.105
*VEGF* −634						
GC	9.1 (7.5–11.3)	1.21 (0.81–1.82)		9.0 (7.0–10.3)	1.14 (0.77–1.68)	
GG/CC	11.7 (9.1–12.9)	1.00	0.356	9.1 (8.0–11.3)	1.00	0.513
*VEGF* +936						
TT	7.8 (1.7–9.1)	3.63 (1.09–12.14)		28.0 (–)	0.34 (0.04–2.76)	
CT/CC	10.2 (9.0–12.4)	1.00	0.036	9.1 (8.3–10.2)	1.00	0.311
*eNOS* +894						
GT	8.9 (6.8–10.2)	1.70 (1.12–2.60)		9.0 (7.4–9.6)	1.06 (0.73–1.54)	
GG/TT	11.9 (9.6–14.1)	1.00	0.013	10.0 (8.3–11.4)	1.00	0.773
*eNOS VNTR*						
4bb	10.9 (9.1–12.9)	0.63 (0.41–0.96)		9.1 (8.3–10.3)	1.09 (0.69–1.74)	
4ab/4aa	9.1 (6.2–11.3)	1.00	0.034	8.9 (6.1–11.6)	1.00	0.708
*eNOS* −786						
CC	12.7 (4.7–14.3)	0.90 (0.52–1.56)		8.9 (4.2–11.5)	1.17 (0.68–2.03)	
CT/TT	9.6 (8.5–11.3)	1.00	0.709	9.1 (8.6–10.3)	1.00	0.572

*Adjusted for CT (FOLFOX4/FOLFIRI), gender, age, *KRAS* status, tumor localization (rectum/colon).

Formal tests of interaction between SNPs and treatment were not significant.

Polymorphisms were also investigated in relation to ORR and OS. For *VEGF* SNPs, only *VEGF* −634 G/C was associated with ORR, a lower rate seen in heterozygous patients compared to the other genotypes (*P* = 0.017) (see Additional file 4). With regard to *eNOS*, *eNOS* +894 G/T was significantly associated with ORR, heterozygous patients showing a lower ORR (42.5%) than the other genotypes (63.1%) (*P* = 0.030) (see Additional file 4). No polymorphism reached the *P* value threshold adjusted for multiple testing. No significant associations were observed between *VEGF* and *eNOS* polymorphisms and ORR in the control group (data not shown).

With regard to OS, patients bearing the *VEGF* −634 GC genotype showed a shorter OS (19.3 months, 95% CI 13.1–22.0) than those with the *VEGF* −634 GG/CC genotype (29.1 months, 95% CI 20.9–33.5), although the difference was not statistically significant (*P* = 0.064) (Table 3). No statistically significant differences were observed in the control group (Table 3). Moreover, patients bearing the *VEGF* +936 TT genotype showed a significantly shorter median OS than *VEGF* +936 TC/CC patients (8.6 months, 95% CI 7.9–13.9 vs 22.7 months, 95% CI 20.5–27.5, respectively, *P* = 0.007; these data remained statistically significant after FDR correction) (Table 3). With regard to *eNOS* polymorphisms and OS, *eNOS* +894 G/T was again found to be associated with outcome. In particular, patients bearing the *eNOS* +894 GT genotype showed a shorter OS (20.1 months, 95% CI 12.0–23.2) compared to *eNOS* +894 GG/TT patients (26.1 months, 95% CI 21.0–33.5, *P* = 0.014) (Table 3). These data remained statistically significant after FDR correction. No statistically significant differences were observed in the control group (Table 3). Finally, *eNOS* VNTR 4a/b was also associated with OS, and patients with a VNTR 4bb genotype showed a longer OS (24.8 months, 95% CI 20.1–34.5) compared to those with the *eNOS* VNTR 4ab/aa genotype (20.6 months, 95% CI 13.7–24.7, *P* = 0.015; not significant after FDR correction) (Table 3). No differences were observed in the control group (Table 3).

**Table 3:** Association between *VEGF* and *eNOS* polymorphisms and overall survival (OS)

Polymorphisms	CT + B			CT		
	Median OS (95% CI)	HR (95% CI)	P*	Median OS (95% CI)	HR (95% CI)	P*
*VEGF* −2578						
AA	26.1 (9.0–34.5)	1.05 (0.61–1.81)		24.0 (14.4–36.7)	1.11 (0.67–1.85)	
CC/CA	21.4 (19.3–27.2)	1.00	0.854	20.8 (19.2–24.5)	1.00	0.671
*VEGF* −1498						
CC	26.1 (10.4–33.1)	1.12 (0.66–1.91)		26.6 (14.4–36.7)	0.95 (0.56–1.59)	
TT/CT	21.8 (19.3-27.2)	1.00	0.664	20.8 (18.8–24.3)	1.00	0.834
*VEGF* −1154						
AA	21.8 (2.3–30.4)	1.18 (0.53–2.63)		20.2 (11.1–29.2)	1.92 (0.96–3.85)	
GA/GG	21.4 (14.6–28.8)	1.00	0.686	25.2 (19.2–29.1)	1.00	0.066
*VEGF* −634						
GC	19.3 (13.1–22.0)	1.54 (0.98–2.44)		20.2 (16.8–24.3)	1.18 (0.76–1.82)	
GG/CC	29.1 (20.9–33.5)	1.00	0.064	24.3 (20.2–29.2)	1.00	0.456
*VEGF* +936						
TT	8.6 (7.9–13.9)	5.48 (1.60–18.8)		28.0 (–)	0.96 (0.11–7.98)	
CT/CC	22.7 (20.5–27.5)	1.00	0.007	21.3 (19.2–25.2)	1.00	0.967
*eNOS* +894						
GT	20.1 (12.0–23.2)	1.80 (1.12–2.89)		24.3 (17.9–28.6)	0.82 (0.53–1.25)	
GG/TT	26.1 (21.0–33.5)	1.00	0.014	21.3 (19.2–26.4)	1.00	0.356
*eNOS VNTR*						
4bb	24.8 (20.1–34.5)	0.54 (0.33–0.89)		23.6 (19.9–28.0)	0.76 (0.46–1.23)	
4ab/4aa	20.6 (13.7–24.7)	1.00	0.015	20.1 (15.0–23.3)	1.00	0.259
*eNOS* −786						
CC	27.2 (14.6–33.5)	0.97 (0.53–1.80)		20.4 (16.0–21.7)	1.40 (0.77–2.55)	
CT/TT	21.3 (16.4–27.4)	1.00	0.931	23.6 (19.9–28.0)	1.00	0.270

*Adjusted for CT (FOLFOX4/FOLFIRI), gender, age, *KRAS* status, tumor localization (rectum/colon).

### Haplotype analysis

Haplotype analysis was performed to evaluate the combined effect of SNPs in the promoter, 5′UTR and 3′UTR of the *VEGF* gene on treatment response. Analysis showed a single block of high linkage disequilibrium formed by three *VEGF* polymorphisms upstream of the coding sequence (promoter and 5′ UTR). Three haplotypes based on *VEGF* −2578 C/A, −1498 C/T and −634 G/C were defined on the basis of the population frequencies of the three SNPs (*VEGF* Haplo1: A–C–G 46.6%; *VEGF* Haplo 2: C–T–C 34%; and *VEGF* Haplo 3: C–T–G 16.5%). None of these haplotypes were significantly associated with clinical outcome. Haplotype analysis of *eNOS* polymorphisms showed a strong linkage disequilibrium between *eNOS* VNTR4a/b and *eNOS* +894 G/T (correlation coefficient, r^2^ = 0.084; Lewontin’s D′ = 0.926) and *eNOS* VNTR 4a/b and *eNOS* −786 T/C (r^2^ = 0.217, D′ = 0.903). Moreover, a weak correlation was found between *eNOS* +894 G/T and *eNOS* −786 T/C polymorphisms (r^2^ = 0.156, D′ = 0.456). Three haplotypes based on *eNOS* VNTR 4a/b and *eNOS* +894 G/T were defined: *eNOS* Haplo 1 (4b-G, 50.2%), *eNOS* Haplo 2 (4b-T, 34.1%) and *eNOS* Haplo 3 (4a-G, 15.3%).

**Fig. 2:**
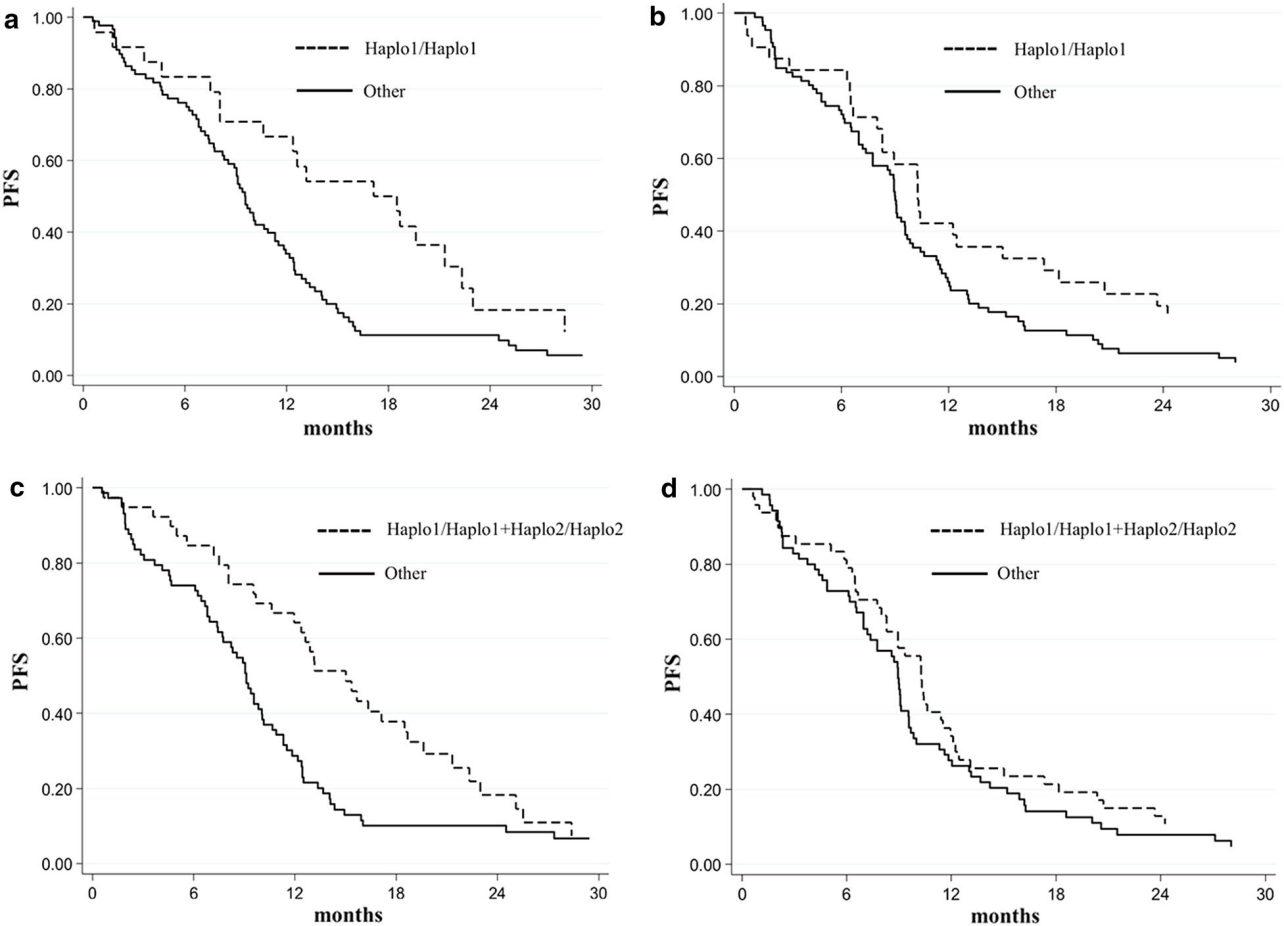
PFS in relation to Haplo1/Haplo1 (*top panels*) or Haplo1/Haplo1 plus Haplo2/Haplo2 (*bottom panels*) genotypes in patients treated with CT + B (**a**, **c**) or CT (**b**, **d**).

In the CT + B arm, patients homozygous for *eNOS* Haplo 1 (*eNOS* Haplo 1/Haplo 1) and the group composed of *eNOS* Haplo 1/Haplo 1 patients and individuals homozygous for Haplo 2 (*eNOS* Haplo 2/Haplo 2) showed a significantly improved outcome compared to the other patients. In particular, a longer median PFS was observed in *eNOS* Haplo1/Haplo1 patients compared to the others in the CT + B group [17.8 months (95% CI 8.1–22.3) vs 9.6 (95% CI 8.3–10.9) (*P* = 0.004)]; this was not observed in the CT only arm (10.3, 95% CI 8.0–15.0, vs. 9.0, 95% CI 7.8–9.6, *P* = 0.123) (Table 4; Fig. 2a, b). More significant results were observed in the CT + B group when *eNOS* Haplo1/Haplo1 and *eNOS* Haplo 2/Haplo 2 patients were combined, with a median PFS of 15.0 months (95% CI 10.6–18.7) compared to 9.1 (95% CI 7.4–10.1) months for those with other genotypes (*P* = 0.001). Both *eNOS* haplotyes reached the *P* value threshold adjusted for FDR correction. No significant differences were observed in the control group (10.3, 95% CI 8.3–11.5, vs 9.0, 95% CI 7.2–9.6, months, *P* = 0.542) (Table 4; Fig. 2c, d).

**Table 4:** Association between *eNOS* Haplotypes and PFS and OS

*eNOS* haplotypes	CT + B				CT			
	n	Median PFS (95% CI)	HR (95% CI)	P*	n	Median PFS (95% CI)	HR (95% CI)	P*
Haplo1/Haplo1	24	17.8 (8.1–22.3)	0.46 (0.27–0.78)		33	10.3 (8.0–15.0)	0.71 (0.46–1.10)	
Other	90	9.6 (8.3–10.9)	1.00	0.004	86	9.0 (7.8–9.6)	1.00	0.123
Haplo2/Haplo2	15	13.1 (7.2–15.7)	0.82 (0.46–1.46)		16	9.9 (5.9–11.5)	1.44 (0.82–2.51)	
Other	99	9.6 (8.3–11.3)	1.00	0.501	107	9.0 (8.3–10.2)	1.00	0.199
Haplo1/Haplo1 + Haplo2/Haplo2	39	15.0 (10.6–18.7)	0.48 (0.30–0.75)		49	10.3 (8.3–11.5)	0.88 (0.60–1.31)	
Other	75	9.1 (7.4–10.1)	1.00	0.001	70	9.0 (7.2–9.6)	1.00	0.542

*eNOS* haplotypes	CT + B				CT			
	n	Median OS (95% CI)	HR (95% CI)	P*	n	Median OS (95% CI)	HR (95% CI)	P*
Haplo1/Haplo1	24	31.6 (19.3–nr)	0.46 (0.24–0.86)		33	23.2 (16.8–39.7)	0.80 (0.49–1.31)	
Other	90	21.3 (15.9–25.2)	1.00	0.016	86	20.8 (18.2–24.5)	1.00	0.379
Haplo2/Haplo2	15	34.5 (13.1–42.9)	0.70 (0.37–1.31)		16	21.3 (14.4–26.4)	1.35 (0.73–2.50)	
Other	99	21.3 (16.4–24.7)	1.00	0.260	107	21.6 (19.2–27.1)	1.00	0.331
Haplo1/Haplo1 + Haplo2/Haplo2	39	34.5 (23.4–38.4)	0.42 (0.25–0.72)		49	21.7 (18.8–29.6)	0.95 (0.61–1.48)	
Other	75	20.5 (14.4–22.7)	1.00	0.002	70	20.8 (18.2–27.1)	1.00	0.829

*Adjusted for CT (FOLFOX4/FOLFIRI), gender, age, *KRAS* status, tumor localization (rectum/colon).

Formal tests of interaction between haplotypes and treatment were not significant.

Moreover, a 71% ORR was observed in Haplo1/Haplo1 and Haplo2/Haplo2 patients compared to 45.9% in patients with other genotypes (*P* = 0.013) (see Additional file 5). We also analyzed *eNOS* haplotypes in relation to OS. Patients with *eNOS* Haplo1/Haplo1 showed a longer median OS (31.6 months, 95% CI 19.3-not reached) than those with other genotypes (21.3, 95% CI 15.9–25.2) (*P* = 0.016), the statistical significance was more evident for *eNOS* Haplo1/Haplo1 and *eNOS* Haplo2/Haplo2 patients (34.5, 95% CI 23.4–38.4, vs 20.5, 95% CI 14.4–22.7, respectively, *P* = 0.002) (Table 4). This latter *eNOS* haplotype passed the FDR correction. No differences were observed within the control group in relation to these different genotypes (Table 4). The results obtained were independent of *KRAS* status.

Interestingly, with regard to the other genotypes, no differences in PFS or OS were observed between patients who were treated or not with B (median PFS 9.1 vs 9.0 months and median OS 20.5 vs 20.8 months, respectively). Conversely, the benefit of B was clearly visible in patients carrying the identified haplotype combination (Table 4).

## Discussion

The present study analyzed *VEGF* and *eNOS* polymorphisms in relation to clinical outcome in patients with metastatic colorectal cancer receiving bevacizumab-based chemotherapy. Although numerous studies have reported significant results regarding *VEGF* polymorphisms, others have failed to show such associations, perhaps because of different study designs. A retrospective study by Loupakis and colleagues [11], hypothesized a role of the *VEGF* −1498 C/T polymorphism as a predictive biomarker of bevacizumab efficacy, showing that patients with a *VEGF* −1498 TT genotype had a worse prognosis. However, the same authors did not confirm this hypothesis in a subsequent prospective study [12] in which only another SNP, VEGFR2 rs12505758, was found to be significantly correlated with PFS. In another retrospective study, *VEGF* −1498 C/T and −2578 A/C were found to be correlated with outcome. In particular, the *VEGF* −2578 CC and −1498 CC genotypes were associated with lower response and hypertension [10]. In other prospective studies, *VEGF* −1154 G/A [9] and *VEGF* −634 G/C [8] were significantly correlated with patient outcome. However, neither study had a chemotherapy only (control) group, making it impossible to reach definitive conclusions.

One strength of our study was the fact that molecular analyses were performed on patients enrolled onto a randomized, prospective phase III multicentre study (ITACa trial) in which two treatment arms were analyzed: chemotherapy plus bevacizumab vs chemotherapy only (highest level of evidence). Our results did not confirm the predictive value of *VEGF* −2578, −1498 and −1154 polymorphisms, whereas *VEGF* −634 was found to be associated with OS. In particular, patients with a heterozygous genotype showed a significantly lower ORR and a lower OS with a trend toward statistical significance. The poorer outcome observed with the heterozygous genotype suggests that the presence of both alleles enhance their negative effect. Moreover, in our case series, the three patients with a *VEGF* +936 TT showed a shorter PFS and a substantially shorter OS. This finding, albeit based on a small number of patients, remained statistically significant after multiple testing corrections and might be worth validating in a larger case series.

The novelty of our work lies in its analysis of *eNOS* polymorphisms in relation to the clinical outcome of patients treated with bevacizumab. Our results show that *eNOS* +894 G/T and *eNOS* VNTR 4a/b were the most interesting polymorphisms. In particular, we identified a specific haplotype (*eNOS* Haplo1/Haplo1, characterised by *eNOS* +894GG and *eNOS* VNTR 4bb) that was significantly associated with improved PFS and OS. Moreover, the combination of *eNOS* Haplo1/Haplo1 and *eNOS* Haplo 2/Haplo2 (characterised by *eNOS* +894TT and *eNOS* VNTR 4bb) accurately identified patients with a better ORR, PFS and OS. Interestingly, no significant associations were found in the group of patients treated with chemotherapy only, reinforcing the predictive value of the haplotypes in relation to bevacizumab efficacy.

*eNOS* is a constitutively expressed gene in endothelium involved in the production of nitric oxide (NO), which plays a central role in maintaining endothelial cell functional integrity, regulating hemodynamics, and establishing collateral circulation [19]. Adequate NO production, consequent to adequate eNOS expression and activity, is essential for preventing thrombotic and atherogenic processes [20]. It has been shown that VEGF inhibition induces a decrease in eNOS expression and thus in NO production [21], and that this phenomenon is linked to the induction of hypertension, one of the most common dose-limiting toxicities of VEGF inhibitors [22]. Previous studies have demonstrated an association between specific *eNOS* polymorphisms and hypertension [14, 23]. A positive correlation between the induction of hypertension and the clinical benefit of bevacizumab has also been observed [13]. Although we did not observe a statistical significance between *eNOS* polymorphisms and hypertension (data not shown), we did find that *eNOS* genotypes associated with a better outcome were also associated with a trend towards higher grade hypertension. Intron 4 *eNOS* VNTR polymorphism plays a role in regulating eNOS expression by acting as an enhancer/repressor and by coding for a 27-nt small RNA which appears to inhibit eNOS expression at the transcriptional level [24–27]. The higher the number of 27-nt repeats, the more 27nt sir-RNA is produced, inhibiting *eNOS* expression. However, the association between *eNOS* VNTR in intron 4 and *eNOS* expression is still a much debated issue [28–30]. Our results showed that patients homozygous for the five repetitions (4bb), who presumably had a lower eNOS expression, showed a better response to bevacizumab. With respect to *eNOS* +894, it has been demonstrated that the +894 TT genotype is associated with lower *eNOS* activity [31–33]. In our study, the haplotype most frequently associated with better PFS and OS was the one homozygous for *eNOS* VNTR 4bb and *eNOS* +894 TT. However, the association with the haplotype homozygous for *eNOS* VNTR 4bb and *eNOS* +894 GG was also significantly associated with outcome. These results suggest that *eNOS* VNTR 4bb is the genotype most strongly correlated with response to bevacizumab. It also implies that, as *eNOS* is not the direct target of bevacizumab, other factors may be involved in the relation between *eNOS* activity and bevacizumab efficacy. In particular, it is possible that the variants are in linkage disequilibrium with other functional variants in the regulatory regions of the *eNOS* gene.

Our study was carried out on 63% (237/376) of the patients enrolled onto the ITACa study, a much higher percentage than those reported by other authors (26% for the AViTA trial and 17% for the AVOREN trial) [34]. This relatively low number of patients did not allow us to reach a sufficient statistical power to test the formal interaction between SNPs and clinical outcome or to consider our results definitively validated. Thus, although the work was based on patients from a randomized clinical trial with a control arm and SNP determinations were prospectively planned and centrally performed, our findings require further validation in independent and larger case series before they can be implemented into clinical practice.

## Conclusions

In conclusion, we identified a haplotype combination of *eNOS* polymorphisms capable of identifying patients who may/will probably benefit from bevacizumab-based chemotherapy. No advantage was observed from the use of bevacizumab in patients not harboring the identified haplotype combination, whereas those carrying the specific genotype showed a significant improvement in ORR, PFS and OS. If confirmed in future studies, this haplotype combination could represent a valid criterion for selecting candidates for treatment with bevacizumab.

## Additional files

Additional file 1: Primer sequences for *VEGF* and *eNOS* SNPs. Additional file 2: Genotype distribution of *VEGF* and e*NOS* polymorphisms. Additional file 3:
**Figure S1.** PFS in relation to *eNOS* +894 (top panels) and VNTR 4ab (bottom panels) polymorphisms in patients treated with CT + B (A, C) or CT alone (B, D). Additional file 4: Correlations between *VEGF* and *eNOS* polymorphisms and overall response rate (ORR) in CT+B arm. Additional file 5: Correlation between *eNOS* haplotypes and ORR.

## Abbreviations

mCRC: metastatic colorectal cancer
*VEGF*: vascular endothelial growth factor
*eNOS*: endothelial nitric oxide synthase
SNPs: single nucleotide polymorphisms
VNTR: variable number tandem repeat
PFS: progression-free survival
ORR: objective response rate
OS: overall survival
mCRC: metastatic colorectal cancer
MoAb: monoclonal antibody
*EGFR*: epidermal growth factor receptor
PD: progression disease
CR: complete response
PR: partial response
SD: stable disease
FFPE: formalin-fixed paraffin- embedded
rs: refSNP
LD: linkage disequilibrium
FDR: false discovery rate
