# Supplementary material for: eNOS polymorphisms as predictors of efficacy of bevacizumab-based chemotherapy in metastatic colorectal cancer: data from a randomized clinical trial
Source: J Transl Med. 2015 Aug 11;13:258. doi: 10.1186/s12967-015-0619-5 (PMC4531503; doi:10.1186/s12967-015-0619-5)
Supplement: Additional file 1: — Primer sequences for VEGF and eNOS SNPs. [file 12967_2015_619_MOESM1_ESM.doc]

| **Additional file 1 Primer sequences for *VEGF* and *eNOS* SNPs** | | | |
| --- | --- | --- | --- |
| **SNPs** | **Primer Sequences** | **Annealing Temperature** | **Amplicon size** |
| *VEGF*-2578C>A | F:5’-AAC-CTA-GCA-CCT-CCA-CCA-AA-3’  R:5’- GCT-GGT-TTC-TGA-CCT-GGC-TA-3’ | 60°C | 268 bp |
| *VEGF*-1498C>T | F:5’-AAG-CCC-ATT-CCC-TCT-TTA-GC-3’  R:5’- CTG-AGA-GCC-GTT-CCC-TCT-TT-3’ | 60°C | 303 bp |
| *VEGF*-1498C>T nested | F:5’ ACA-GGG-AAG-CTG-GGT-GAA-T-3’  R:5’ CTG-AGA-GCC-GTT-CCC-TCT-TT-3’ | 58°C | 235 bp |
| *VEGF*-1154G>A | F:5’-TTT-TCA-GGC-TGT-GAA-CCT-TG-3’  R:5’- ACG-ACC-TCC-GAG-CTA-CCC-3’ | 62°C | 264 bp |
| *VEGF*-1154G>A nested | F:5’ TTT-TCA-GGC-TGT-GAA-CCT-TG-3’  R:5’ GAT-CCT-CCC-CGC-TAC-CAG-3’ | 60°C | 227 bp |
| *VEGF*-634C>G | F:5’-GGA-TTT-TGG-AAA-CCA-GCA-GA-3’  R:5’- CTG-TCT-GTC-TGT-CCG-TCA-GC3’ | 62°C | 224 bp |
| *VEGF*+936C>T | F:5’-ACA-CCA-TCA-CCA-TCG-ACA-GA-3’  R:5’- CAG-GAA-TCC-CAG-AAA-TAA-AAC-TC-3’ | 58°C | 226 bp |
| *eNOS*+894G>T | F:5’-AAG-GCA-GGA-GAC-AGT-GGA-TG-3’  R:5’- GTT-GGG-GTG-TGG-GAT-CAG-3’ | 64°C | 319 bp |
| *eNOS* VNTR 27bp | F:5’- AAA-CTG-TGG-GGG-AGA-TCC-TT-3’  R:5’-GGG-CAG-CTT-GCT-TCT-CTT-AG-3’ | 62°C | 544 bp |
| F= forward primer; R= reverse primer | | | |
