# Supplementary material for: eNOS polymorphisms as predictors of efficacy of bevacizumab-based chemotherapy in metastatic colorectal cancer: data from a randomized clinical trial
Source: J Transl Med. 2015 Aug 11;13:258. doi: 10.1186/s12967-015-0619-5 (PMC4531503; doi:10.1186/s12967-015-0619-5)
Supplement: Additional file 2: — Genotype distribution of VEGF and eNOS polymorphisms. [file 12967_2015_619_MOESM2_ESM.doc]

| **Additional file 2 Genotype distribution of *VEGF* and e*NOS* polymorphisms** | | | |
| --- | --- | --- | --- |
|  | **CT + B**  **n (%)** | **CT**  **n (%)** | **Total**  **n (%)** |
| ***VEGF* polymorphisms** |  |  |  |
| ***VEGF*-2578 C>A** |  |  |  |
| C/C | 29 (25.5) | 34 (27.6) | 63 (26.6) |
| C/A | 60 (52.6) | 60 (48.8) | 120 (50.6) |
| A/A | 25 (21.9) | 29 (23.6) | 54 (22.8) |
| ***VEGF*-1498 C>T** |  |  |  |
| C/C | 26 (22.8) | 29 (23.6) | 55 (23.2) |
| C/T | 60 (52.6) | 57 (46.3) | 117 (49.4) |
| T/T | 28 (24.6) | 37 (30.1) | 65 (27.4) |
| ***VEGF*-1154 G>A** |  |  |  |
| G/G | 38 (48.7) | 33 (44.0) | 71 (46.4) |
| G/A | 29 (37.2) | 31 (41.3) | 60 (39.2) |
| A/A | 11 (14.1) | 11 (14.7) | 22 (14.4) |
| ***VEGF*-634 G>C** |  |  |  |
| G/G | 47 (41.2) | 54 (43.9) | 101 (42.6) |
| G/C | 49 (43.0) | 52 (42.3) | 101 (42.6) |
| C/C | 18 (15.8) | 17 (13.8) | 35 (14.8) |
| ***VEGF*+936 C>T** |  |  |  |
| C/C | 78 (68.4) | 95 (77.9) | 173 (73.3) |
| C/T | 33 (29.0) | 26 (21.3) | 59 (25.0) |
| T/T | 3 (2.6) | 1 (0.8) | 4 (1.7) |
| n.e. | - | 1 | 1 |
| ***eNOS* polymorphisms** |  |  |  |
| ***eNOS*+894 G>T** |  |  |  |
| G/G | 51 (44.7) | 53 (43.1) | 104 (43.9) |
| G/T | 48 (42.1) | 54 (43.9) | 102 (43.0) |
| T/T | 15 (13.2) | 16 (13.2) | 31 (13.1) |
| ***eNOS* VNTR 27bp** |  |  |  |
| b/b | 76 (66.7) | 86 (72.3) | 162 (69.5) |
| a/b | 37 (32.4) | 31 (26.0) | 68 (29.2) |
| a/a | 1 (0.9) | 2 (1.7) | 3 (1.3) |
| NE | - | 4 | 4 |
| ***eNOS*-786 T>C** |  |  |  |
| T/T | 34 (29.8) | 46 (37.7) | 80 (33.9) |
| T/C | 62 (54.4) | 55 (45.1) | 117 (49.6) |
| C/C | 18 (15.8) | 21 (17.2) | 39 (16.5) |
| n.e. | - | 1 | 1 |

n.e., not evaluable
