# Supplementary figures and images for: eNOS polymorphisms as predictors of efficacy of bevacizumab-based chemotherapy in metastatic colorectal cancer: data from a randomized clinical trial
Source: J Transl Med. 2015 Aug 11;13:258. doi: 10.1186/s12967-015-0619-5 (PMC4531503; doi:10.1186/s12967-015-0619-5)

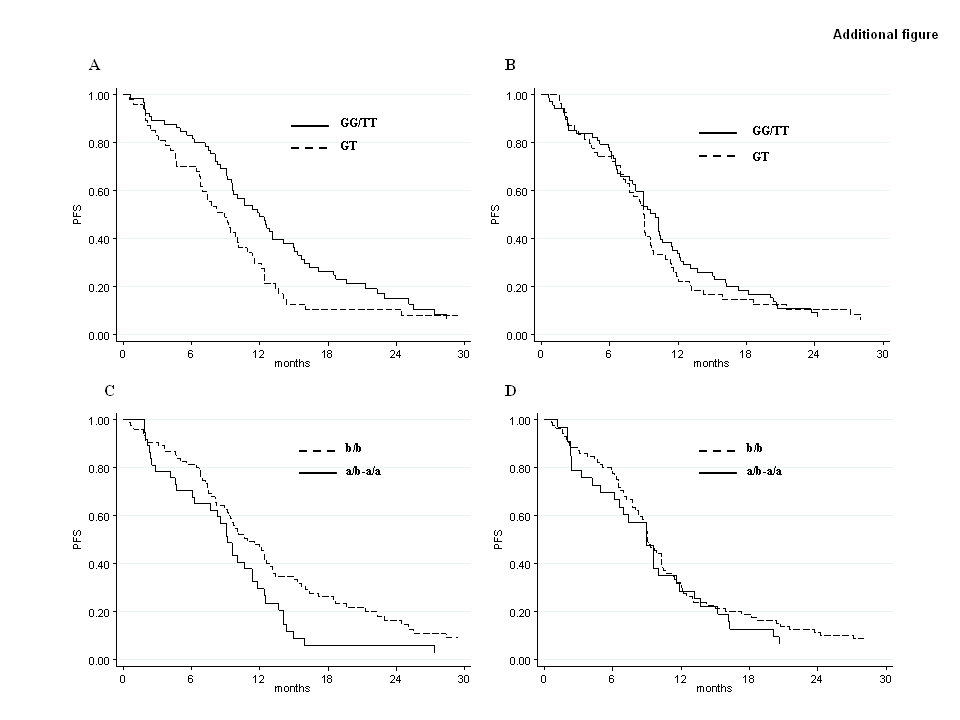

Supplement: Additional file 3: — Figure S1. PFS in relation to eNOS +894 (top panels) and VNTR 4ab (bottom panels) polymorphisms in patients treated with CT + B (A, C) or CT alone (B, D). [file 12967_2015_619_MOESM3_ESM.tiff]
