# Supplementary material for: eNOS polymorphisms as predictors of efficacy of bevacizumab-based chemotherapy in metastatic colorectal cancer: data from a randomized clinical trial
Source: J Transl Med. 2015 Aug 11;13:258. doi: 10.1186/s12967-015-0619-5 (PMC4531503; doi:10.1186/s12967-015-0619-5)
Supplement: Additional file 4: — Correlations between VEGF and eNOS polymorphisms and overall response rate (ORR) in CT+B arm. [file 12967_2015_619_MOESM4_ESM.doc]

| **Additional file 4 Correlations between *VEGF* and *eNOS* polymorphisms and overall response rate (ORR) in CT+B arm** | | | | | |
| --- | --- | --- | --- | --- | --- |
| **SNPs** | **Patients**  **n** | **CR/PR**  **n (%)** | **SD/PD**  **n (%)** | ***P**** | **Odds ratio**  **(95% IC)** |
| ***VEGF* -2578** |  |  |  |  |  |
| AA | 25 | 15 (60.0) | 10 (40.0) | 0.305 | 1.64 (0.64-4.25) |
| CC/CA | 87 | 46 (52.9) | 41 (47.1) |  | 1.00 |
| ***VEGF* -1498** |  |  |  |  |  |
| CC | 26 | 15 (57.7) | 11 (42.3) | 0.515 | 1.36 (0.54-3.40) |
| TT/CT | 86 | 46 (53.5) | 40 (46.5) |  | 1.00 |
| ***VEGF* -1154** |  |  |  |  |  |
| GG | 38 | 23 (60.5) | 15 (39.5) | 0.914 | 0.95 (0.37-2.45) |
| GA/AA | 34 | 21 (61.8) | 13 (38.2) |  | 1.00 |
| ***VEGF* -634** |  |  |  |  |  |
| GC | 47 | 20 (42.5) | 27 (57.5) | **0**.**017** | 0.37 (0.16-0.84) |
| GG/CC | 65 | 41 (63.1) | 24 (36.9) |  | 1.00 |
| ***VEGF* +936** |  |  |  |  |  |
| TT | 3 | 1 (33.3) | 2 (66.7) | 0.317 | 0.28 (0.02-3.36) |
| CT/CC | 109 | 60 (55.1) | 49 (44.9) |  | 1.00 |
| ***eNOS* +894** |  |  |  |  |  |
| GT | 47 | 20 (42.5) | 27 (57.5) | **0**.**030** | 0.41 (0.18-0.91) |
| GG/TT | 65 | 41 (63.1) | 24 (36.9) |  | 1.00 |
| ***eNOS* VNTR** |  |  |  |  |  |
| bb | 74 | 44 (59.5) | 30 (40.5) | 0183 | 1.74 (0.77-3.95) |
| ab/aa | 38 | 17 (44.7) | 21 (55.3) |  | 1.00 |
| ***eNOS*-786** |  |  |  |  |  |
| CC | 18 | 8 (44.4) | 10 (55.6) | 0.578 | 0.74 (0.25-2.14) |
| CT/TT | 94 | 53 (56.4) | 41 (43.6) |  | 1.00 |
| *Adjusted for CT (FOLFOX4/FOLFIRI), gender, age, *KRAS* status, tumor localization (rectum/colon) | | | | | |
