# Supplementary material for: eNOS polymorphisms as predictors of efficacy of bevacizumab-based chemotherapy in metastatic colorectal cancer: data from a randomized clinical trial
Source: J Transl Med. 2015 Aug 11;13:258. doi: 10.1186/s12967-015-0619-5 (PMC4531503; doi:10.1186/s12967-015-0619-5)
Supplement: Additional file 5: — Correlation between eNOS haplotypes and ORR. [file 12967_2015_619_MOESM5_ESM.doc]

| **Additional file 5 Correlation between *eNOS* haplotypes and ORR** | | | | | |
| --- | --- | --- | --- | --- | --- |
| ***eNOS* haplotypes** | **Patients**  **n (%)** | **CR/PR**  **n (%)** | **SD/PD**  **n (%)** | ***P**** | **Odds ratio**  **(95% CI)** |
| **CT + B** |  |  |  |  |  |
| Haplo1/Haplo1 | 23 | 15 (65.2) | 8 (34.8) |  | 1.75 (0.65-4.73) |
| Other | 89 | 46 (51.7) | 43 (48.3) | 0.267 | 1.00 |
|  |  |  |  |  |  |
| Haplo2/Haplo2 | 15 | 12 (80.0) | 3 (20.0) |  | 4.18 (1.07-16.39) |
| Other | 97 | 49 (50.5) | 48 (49.5) | 0.040 | 1.00 |
|  |  |  |  |  |  |
| Haplo1/Haplo1+Haplo2/Haplo2 | 38 | 27 (71.0) | 11 (29.0) |  | 3.09 (1.27-7.51) |
| Other | 74 | 34 (45.9) | 40 (54.1) | 0.013 | 1.00 |
| **CT** |  |  |  |  |  |
| Haplo1/Haplo1 | 32 | 19 (59.4) | 13 (40.6) |  | 1.61 (0.66-3.93) |
| Other | 86 | 45 (52.3) | 41 (47.7) | 0.297 | 1.00 |
|  |  |  |  |  |  |
| Haplo2/Haplo2 | 16 | 8 (50.0) | 8 (50.0) |  | 0.84 (0.27-2.65) |
| Other | 106 | 57 (53.8) | 49 (46.2) | 0.773 | 1.00 |
|  |  |  |  |  |  |
| Haplo1/Haplo1+Haplo2/Haplo2 | 48 | 27 (56.2) | 21 (43.8) |  | 1.33 (0.60-2.95) |
| Other | 70 | 37 (52.9) | 33 (47.1) | 0.486 | 1.00 |
| *Adjusted for CT (FOLFOX/FOLFIRI), gender, age, *KRAS* status, tumor localization (rectum/colon)  Haplo1/ Haplo1: patients homozygous for VNTR 4bb/+894 GG; Haplo2/Haplo2: patients homozygous for VNTR 4bb/+894 TT | | | | | |
